# Supplementary figures and images for: Identification of the reporter gene combination that shows high contrast for cellular level MRI
Source: PLoS One. 2024 Feb 1;19(2):e0297273. doi: 10.1371/journal.pone.0297273 (PMC10833543; doi:10.1371/journal.pone.0297273)

200

100

50

25

12.5

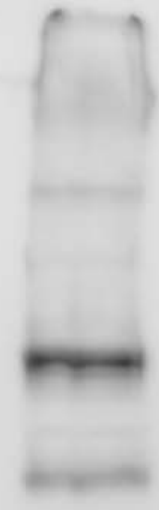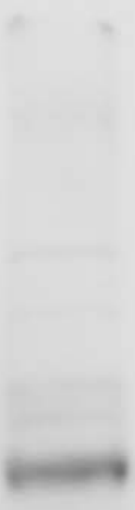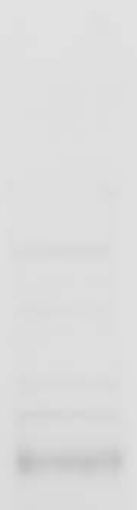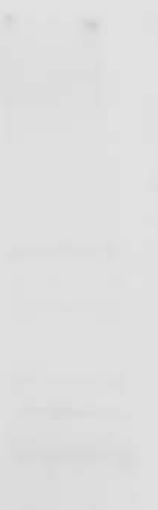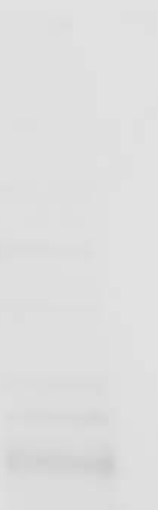

1

1

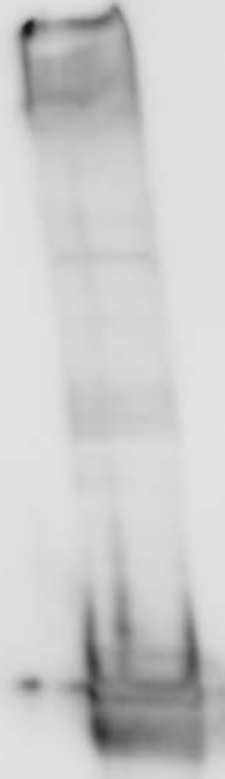

X DMT1 X FnM6A mms6

X

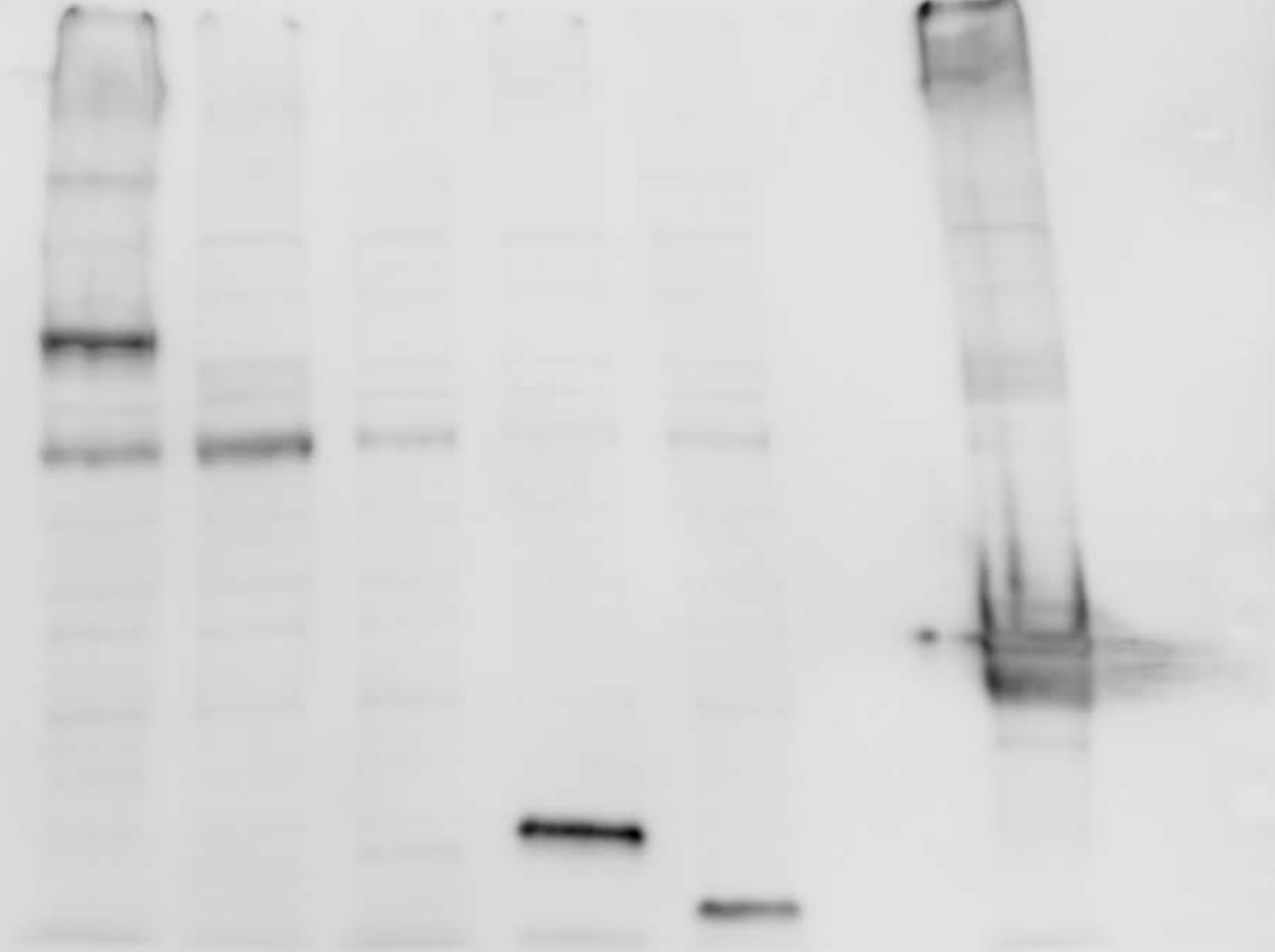

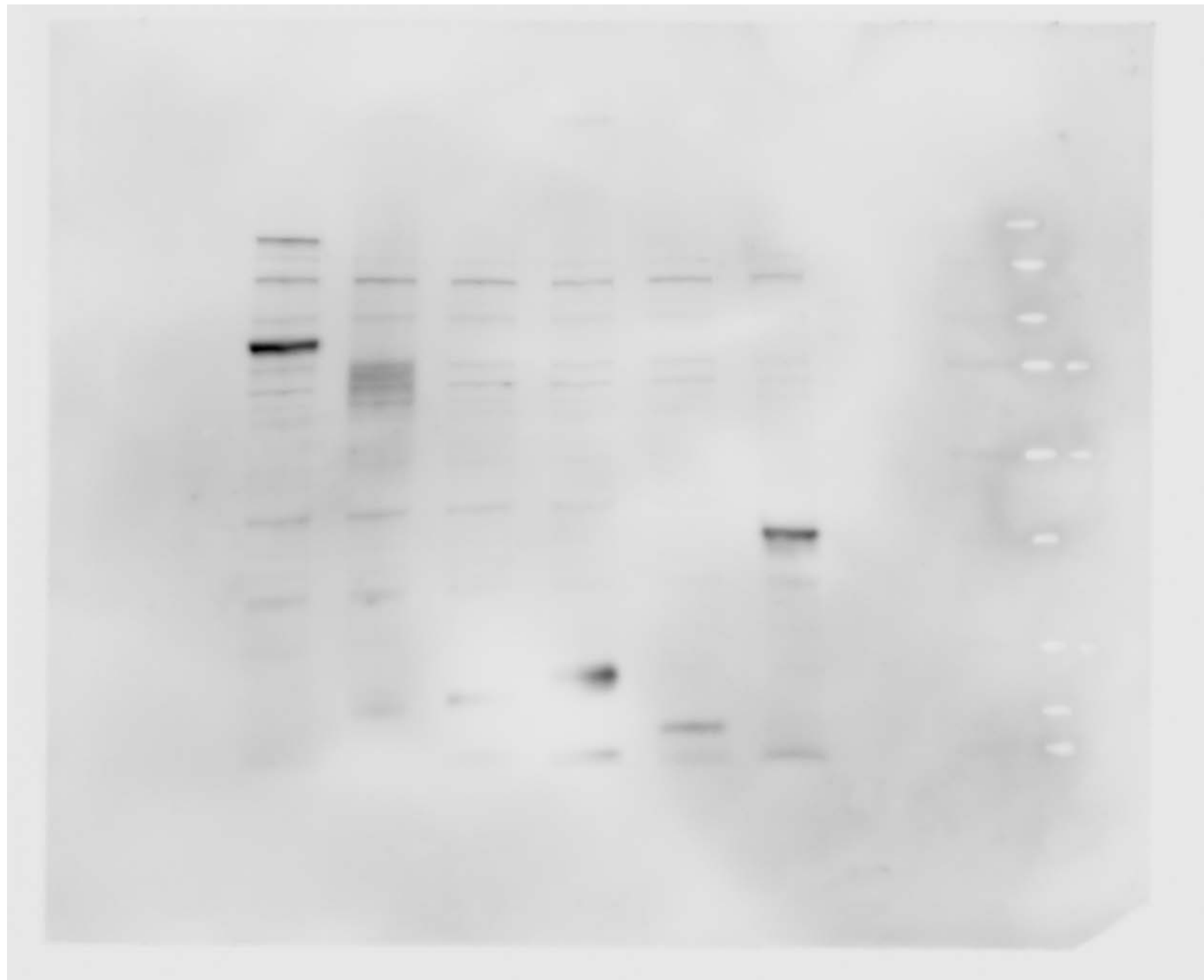

TfR

X

X

Fn

X

X

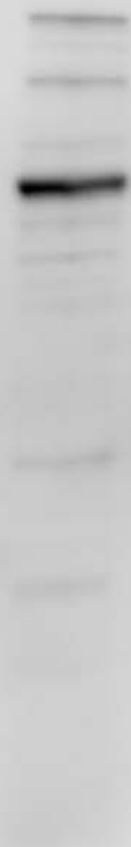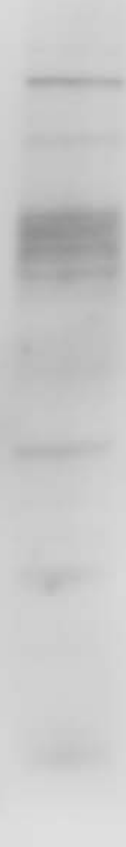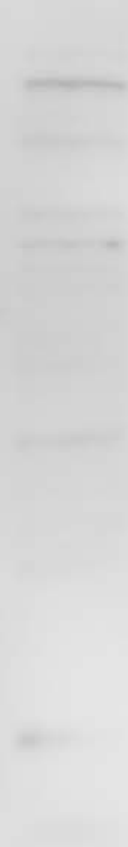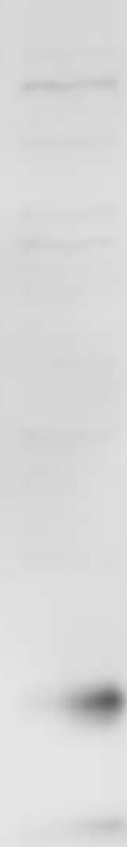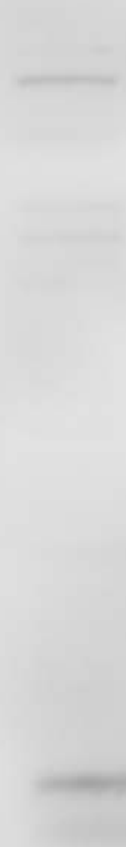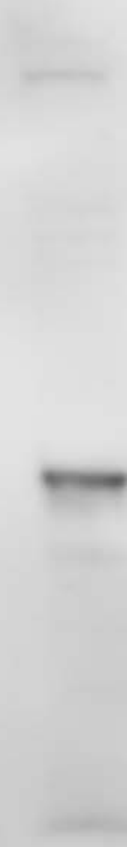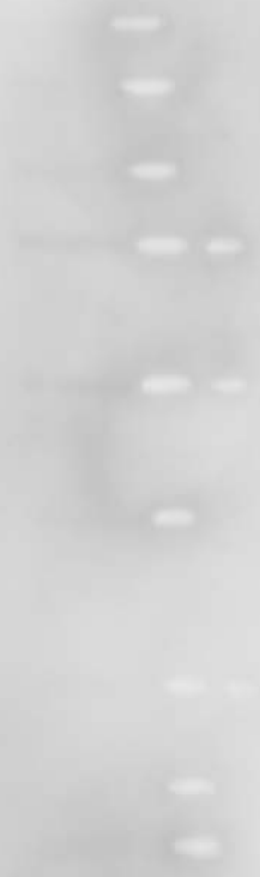

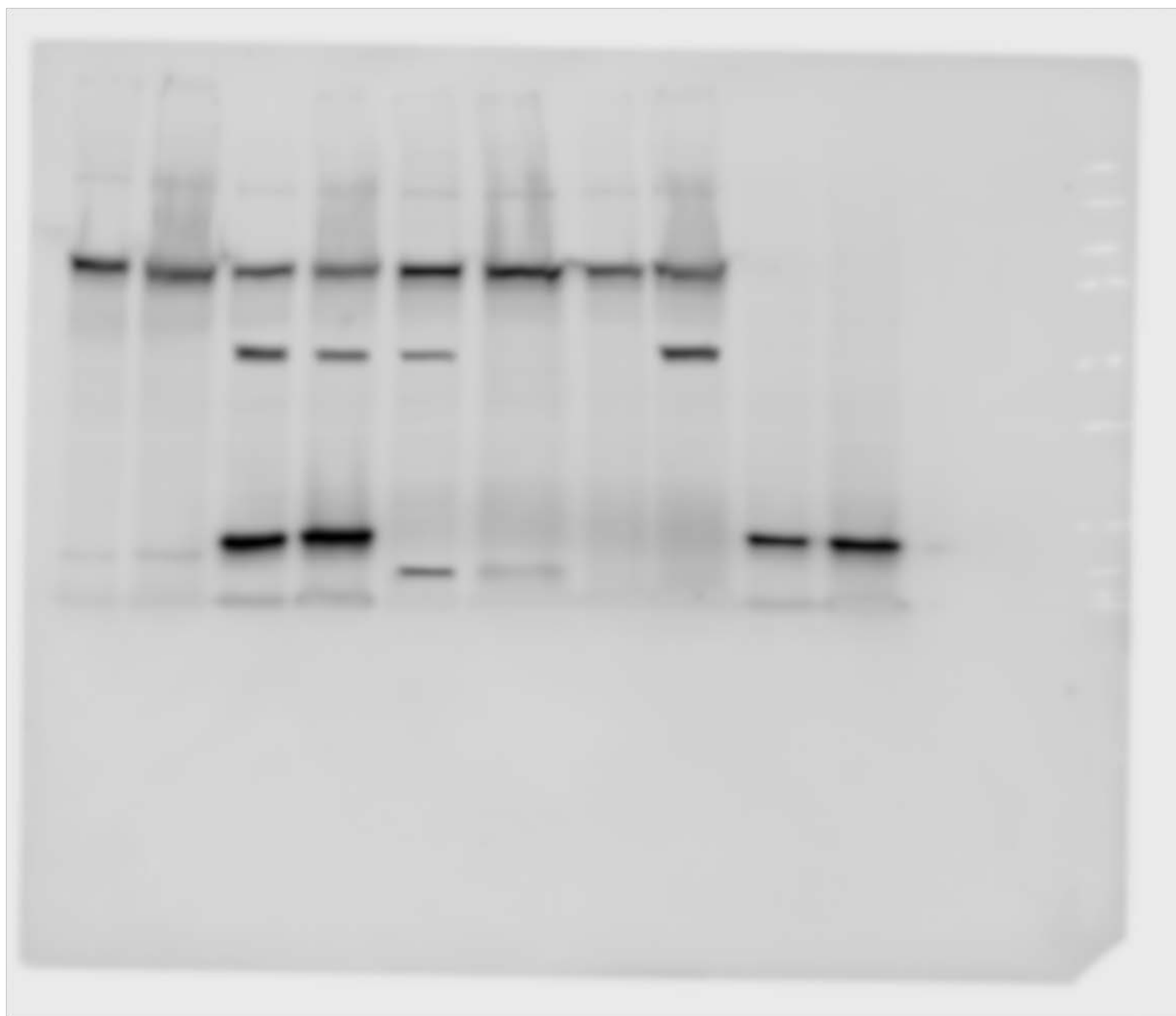

X X (B) (A) (C) X X (D) X (E)

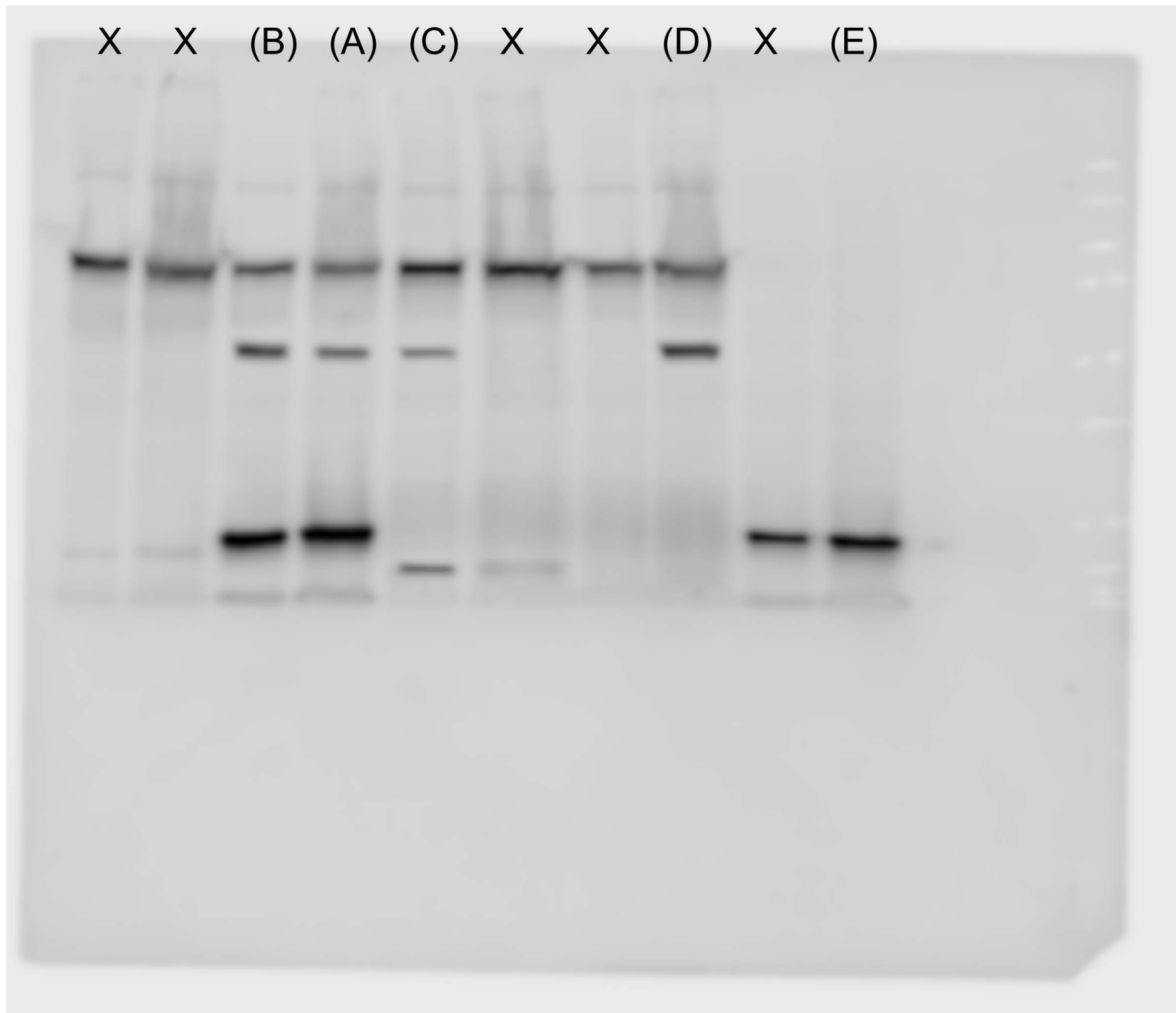

Supplement: S1 Raw images — (PDF) [file pone.0297273.s001.pdf]
