# Supplementary material for: Identification of the reporter gene combination that shows high contrast for cellular level MRI
Source: PLoS One. 2024 Feb 1;19(2):e0297273. doi: 10.1371/journal.pone.0297273 (PMC10833543; doi:10.1371/journal.pone.0297273)
Supplement: Attachment — Submitted filename: Response_to_Reviewers.docx [file pone.0297273.s003.docx]

December 28, 2023

Kazunori Nagasawa

Academic Editor

PLOS ONE

Dear Editor and Reviewers:

We wish to re-submit the manuscript titled “Identification of the reporter gene combination that shows high contrast for cellular level MRI.” The manuscript ID is PONE-D-23-27098R1.

We thank you and the reviewers for your thoughtful comments. The manuscript has benefited from your suggestions, and I would like to address your comments in the following manner. Once again, I am grateful for your valuable insights.

The reference section in the manuscript has been rechecked and the necessary changes have been made in accordance with your suggestions. We hope we have met your intentions. The modified sections are highlighted in “Revised Manuscript with Track Changes” file.

Dear Editor

>>> Please review your reference list to ensure that it is complete and correct. If you have cited papers that have been retracted, please include the rationale for doing so in the manuscript text, or remove these references and replace them with relevant current references. Any changes to the reference list should be mentioned in the rebuttal letter that accompanies your revised manuscript. If you need to cite a retracted article, indicate the article’s retracted status in the References list and also include a citation and full reference for the retraction notice.

Thank you for your comment. We rechecked our reference list and corrected them. We have not removed or added references, only modified some statements. The corrected reference list is as follows:

1.	Blasberg RG, Tjuvajev JG. Molecular-genetic imaging: current and future perspectives. J Clin Invest. 2003;111(11): 1620–1629. doi:10.1172/JCI18855

2.	Shimomura O. Discovery of green fluorescent protein (GFP) (Nobel Lecture). Angew Chem Int Ed Engl. 2009;48(31): 5590–5602. doi:10.1002/anie.200902240

3.	Iyer S, Arindkar S, Mishra A, Manglani K, Kumar JM, Majumdar SS, et al. Development and Evaluation of Transgenic Nude Mice Expressing Ubiquitous Green Fluorescent Protein. Mol Imaging Biol. 2015;17(4): 471–478. doi:10.1007/s11307-014-0821-5

4.	Hall MP, Unch J, Binkowski BF, Valley MP, Butler BL, Wood MG, et al. Engineered luciferase reporter from a deep sea shrimp utilizing a novel imidazopyrazinone substrate. ACS Chem Biol. 2012;7(11): 1848–1857. doi:10.1021/cb3002478.

5.	Zarychta-Wiśniewska W, Burdzinska A, Zagozdzon R, Dybowski B, Butrym M, Gajewski Z, et al. In vivo imaging system for explants analysis-A new approach for assessment of cell transplantation effects in large animal models. PLOS ONE. 2017;12(9): e0184588. doi:10.1371/journal.pone.0184588

6.	Li Y, Jiao Q, Xu H, Du X, Shi L, Jia F, et al. Biometal Dyshomeostasis and Toxic Metal Accumulations in the Development of Alzheimer’s Disease. Front Mol Neurosci. 2017;10: 339. doi:10.3389/fnmol.2017.00339

7.	Aisen P. Transferrin receptor 1. Int J Biochem Cell Biol. 2004;36(11): 2137–2143. doi:10.1016/j.biocel.2004.02.007

8.	Andrews NC. The iron transporter DMT1. Int J Biochem Cell Biol. 1999;31(10): 991–994. doi:10.1016/S1357-2725(99)00065-5

9.	Lewis CM, Graves SA, Hernandez R, Valdovinos HF, Barnhart TE, Cai W, et al. ^52^Mn production for PET/MRI tracking of human stem cells expressing divalent metal transporter 1 (DMT1). Theranostics. 2015;5(3): 227–239. doi:10.7150/thno.10185

10.	Ono K, Fuma K, Tabata K, Sawada M. Ferritin reporter used for gene expression imaging by magnetic resonance. Biochem Biophys Res Commun. 2009;388(3): 589–594. doi:10.1016/j.bbrc.2009.08.055

11.	Yang C, Tian R, Liu T, Liu G. MRI Reporter Genes for Noninvasive Molecular Imaging. Molecules. 2016;21(5):580. doi:10.3390/molecules21050580

12.	Galloway JM, Arakaki A, Masuda F, Tanaka T, Matsunaga T, Staniland SS. Magnetic bacterial protein Mms6 controls morphology, crystallinity and magnetism of cobalt-doped magnetite nanoparticles in vitro. J. Mater. Chem., 2011;21: 15244-15254. doi:10.1039/C1JM12003D

13.	Graham FL, Smiley J, Russell WC, Nairn R. Characteristics of a human cell line transformed by DNA from human adenovirus type 5. J Gen Virol. 1977;36(1): 59–74. doi:10.1099/0022-1317-36-1-59

14.	Yuan J, Xu WW, Jiang S, Yu H, Fai Poon HF. The Scattered Twelve Tribes of HEK293. Biomed Pharmacol J. 2018;11(2):621–623. Available from: http://biomedpharmajournal.org/?p=20696

15.	Radoul M, Lewin L, Cohen B, Oren R, Popov S, Davidov G, et al. Genetic manipulation of iron biomineralization enhances MR relaxivity in a ferritin-M6A chimeric complex. Sci Rep. 2016;6: 26550. doi:10.1038/srep26550

16.	Kurien BT, Scofield RH. Western blotting: an introduction. Methods Mol Biol. 2015;1312: 17–30. doi:10.1007/978-1-4939-2694-7_5

17.	Towbin H, Staehelin T, Gordon J. Electrophoretic transfer of proteins from polyacrylamide gels to nitrocellulose sheets: procedure and some applications. Proc Natl Acad Sci U S A. 1979;76(9): 4350–4354. doi:10.1073/pnas.76.9.4350

18.	Dou Y, Lin Y, Wang TY, Wang XY, Jia YL, Zhao CP. The CAG promoter maintains high-level transgene expression in HEK293 cells. FEBS Open Bio. 2021;11(1): 95–104. doi:10.1002/2211-5463.13029

19.	Einhauer A, Jungbauer A. The FLAG peptide, a versatile fusion tag for the purification of recombinant proteins. J Biochem Biophys Methods. 2001;49(1-3): 455–465. doi:10.1016/s0165-022x(01)00213-5

20.	Dohi T, Murashige K. A Micro Saddle Coil with Switchable Sensitivity for Local High-Resolution Imaging of Luminal Tissue. Micromachines. 2016;7(4): 67. doi:10.3390/mi7040067

Thank you again for your valuable comments and for giving us the opportunity to improve our manuscript. We have worked hard to incorporate your input and hope that these corrections will make you more receptive to our submission.

Sincerely,

Corresponding Auther:

Junichi Hata

Phone: +81-03-3819-1211 (extension; 410), Email: [j-hata@tmu.ac.jp](mailto:j-hata@tmu.ac.jp),

Graduate School of Human Health Sciences, Tokyo Metropolitan University,

7-2-10 Higashi-Ogu, Arakawa-ku, Tokyo, Japan 116-8551

First Auther:

Naoya Hayashi

Department of Radiological Sciences, Graduate School of Human Health Sciences, Tokyo Metropolitan University

7-2-10 Higashi-Ogu, Arakawa-ku, Tokyo, Japan 116-8551

Tel: 03-3819-1211

E-mail: n.hayashi731@gmail.com
